# α-Synuclein-Targeted Immunotherapies in Parkinson’s Disease: In Silico, In Vitro and Clinical Perspectives

**DOI:** 10.3390/molecules31122036

**Published:** 2026-06-10

**Authors:** Tatiane B. Santos, Tatiane de O. X. Machado, Pedro Henrique S. Rodrigues, Willamys S. Correa, Helena A. C. Kodel, Klebson S. Santos, Margarete Z. Gomes

**Affiliations:** 1 Graduate Program in Biosciences and Health, Northeast Biotechnology Network (RENORBIO), Tiradentes University, Farolândia Campus, Av. Murilo Dantas, 300, Aracaju 49032-490, SE, Brazil; tatiane.bdos@souunit.com.br; 2 Laboratory of Morphology and Experimental Pathology, Institute of Technology and Research (ITP), Av. Murilo Dantas, 300, Aracaju 49032-490, SE, Brazil; pedrohenri.biomed@gmail.com (P.H.S.R.); willamys.souza@souunit.com.br (W.S.C.); helenakodel@gmail.com (H.A.C.K.); 3 Department of Agroindustry, Federal Institute of Sertão Pernambucano, Campus Petrolina Zona Rural, PE 647, Km 22, PISNC N4, Petrolina 56302-970, PE, Brazil; tatiane.machado@ifsertao-pe.edu.br; 4 Center for Study on Colloidal Systems (NUESC), Institute of Technology and Research (ITP), Av. Murilo Dantas, 300, Aracaju 49032-490, SE, Brazil

**Keywords:** α-synuclein, immunotherapy, Parkinson’s disease

## Abstract

α-synuclein (α-syn) aggregation in dopaminergic neurons is a central event in Parkinson’s disease (PD) pathogenesis. Immunotherapeutic strategies targeting α-syn, including passive and active approaches, aim to inhibit aggregation, propagation, and toxicity of pathological species while promoting their clearance via immune mechanisms. This review summarizes α-syn directed immunotherapies evaluated in in silico, in vitro, and in vivo models, as well as early phase clinical trials, focusing on how epitope selection and antibody formats influence efficacy, safety, and target engagement. Data on monoclonal antibody, peptide, and protein-based vaccines, and structure-guided immunogens were analyzed, integrating behavioral, neuropathological, proteomic, and structural outcomes alongside biomarker development for α-syn species in cerebrospinal fluid and peripheral compartments. Clinical evidence indicates that several candidates induce sustained anti-α-syn antibody responses with acceptable safety profiles and signs of pharmacodynamic engagement, including reductions in free or oligomeric α-syn. However, consistent long-term clinical benefits remain unproven, highlighting the gap between preclinical success and disease modification in humans. Advances in structural biology and proteomics support rational epitope selection and improved immunogen design, reinforcing α-syn-targeted immunotherapy as a promising yet experimental strategy for PD, and highlighting the need for mechanistically oriented, biomarker-driven clinical trials initiated in well-characterized prodromal and early-stage cohorts.

## 1. Introduction

Parkinson’s disease (PD) is a progressive neurodegenerative pathology defined by the systemic proteotoxicity of misfolded α-synuclein (α-syn) isoforms [1]. Although PD is clinically diagnosed by the cardinal motor impairments of bradykinesia, resting tremor, and postural instability, its etiology involves an extensive prodromal phase characterized by cellular dyshomeostasis that precedes the loss of dopaminergic neurons in the substantia nigra pars compacta (SNpc) [2,3,4,5,6]. The transition from motor symptoms to cognitive decline reflects the stereotypical propagation of α-syn pathology from the brainstem to neocortical regions, driving escalating functional morbidity [5,7,8,9].

Standard pharmacological interventions, notably levodopa, remain limited to symptomatic management and fail to attenuate the underlying neurodegenerative trajectory. Despite their efficacy in restoring dopaminergic tonus, these therapies do not confer disease-modifying or neuroprotective effects [7,10,11,12]. This therapeutic impasse underscores the necessity of addressing the multifactorial pathogenesis of PD, which integrates mitochondrial bioenergetic failure, oxidative stress, and chronic neuroinflammation. Dopaminergic neurons exhibit an intrinsic selective vulnerability, exacerbated by high metabolic demands and L-type Ca^2^ channel kinetics, which facilitate the deleterious accumulation of reactive oxygen species (ROS) [13,14,15].

The molecular landscape of PD is further defined by genetic predispositions, specifically mutations in *PARK* genes (e.g., *PINK1*, *Parkin*, *DJ-1*), and the collapse of the autophagy–lysosome pathway (ALP). These defects impair the clearance of aggregated α-syn, triggering a self-perpetuating cascade of synaptic dysfunction and microglia-mediated neuroinflammation [13,14,15,16,17,18]. Given its capacity for prion-like templating and structural polymorphism, α-syn is now recognized as a central orchestrator of the biological and clinical heterogeneity inherent to PD [1,19,20].

Recent advancements in high-throughput multi-omics—encompassing genomics, transcriptomics, and proteomics—have facilitated the identification of peripheral biomarkers and elucidated the transcriptomic signatures associated with PD progression [21,22,23,24,25]. Specifically, the focus of disease-modifying research has pivoted toward α-syn-targeted immunotherapies. Strategies currently in clinical pipelines, including active immunization (e.g., UB-312) and passive therapies using monoclonal antibodies (e.g., prasinezumab), aim to intercept the extracellular seeding and intracellular toxicity of α-syn [25,26,27].

This review aims to present the current state of therapeutic approaches for PD, with an emphasis on emerging α-syn-targeted immunotherapy strategies. Specifically, we discuss their mechanisms of action, potential benefits, and clinical limitations, while exploring how the integration of proteomic and structural data may guide the development and validation of more effective candidates in preclinical and clinical studies.

## 2. Parkinson’s Disease

PD was first described in 1817 by James Parkinson in his seminal monograph *An Essay on the Shaking Palsy*. Since then, it has become one of the most extensively studied neurodegenerative disorders. The primary motor manifestations of the disease, initially identified through clinical observations of patients typically presenting between 50 and 60 years of age, were later more comprehensively characterized by Jean-Martin Charcot in 1872 [2,3,7,28,29,30,31].

Currently, PD is recognized as a progressive neurodegenerative condition, marked by motor symptoms such as bradykinesia, resting tremor, rigidity, and postural instability, and non-motor symptoms, including cognitive, psychiatric, and gastrointestinal changes, and pre-motor manifestations such as anosmia and sleep disorders [7]. Diagnosis usually occurs in advanced stages, when more than 50% of dopaminergic neurons have already been lost [10].

Pharmacological treatment is primarily based on levodopa, considered the gold standard, along with carbidopa, anticholinergics, and MAO-B and COMT inhibitors. However, these drugs are associated with significant adverse effects, such as dyskinesias [10,32,33]. Despite therapeutic advances, the prevalence of PD continues to increase globally, with projections of significant growth until 2050, especially in individuals over 50 years of age and in males [2,3].

From a molecular standpoint, mutations in the *SNCA* gene are associated with the pathogenesis of PD, favoring α-syn aggregation and the formation of Lewy bodies, although they account for only a small fraction of sporadic and familial cases [34,35]. Given the limitations of current therapies, immunotherapy has emerged as a promising strategy to leverage the interaction between α-syn and the immune system, thereby modulating neuroinflammation and potentially arresting disease progression [36].

### 2.1. Etiology of Parkinson’s Disease

Research on PD has provided valuable insights into its multifactorial etiology; however, the disease remains incompletely understood and is therefore considered idiopathic. As mentioned previously, advanced age remains the main risk factor for PD, although understanding of the roles of genetic and environmental factors has grown considerably. Genes associated with PD include *SNCA*, *Parkin/PARK2*, *UCHL1*, *PINK1*, *DJ1/PARK7*, and *LRRK2*, which are linked to both familial and sporadic forms of the disease [37].

Collectively, mutations or functional alterations in these PD-associated genes converge on disturbed proteostasis, including impaired ALP activity, which limits the clearance of misfolded α-syn species and accelerates the accumulation of toxic aggregates [13,14,15,18]. Environmental factors, such as exposure to toxins including 1-methyl-4-phenyl-1,2,3,6-tetrahydropyridine (MPTP), paraquat, and rotenone, are associated with an increased risk of PD, while the role of heavy metals remains inconclusive. The interaction between genetic and environmental factors remains a central focus of research, aimed at understanding the disease’s etiology and developing therapeutic or preventive strategies targeting pathological α-syn [38,39,40,41,42,43].

### 2.2. Genetic, Structural, and Pathogenic Aspects of α-Syn in Parkinson’s Disease

Among the genes associated with *PD*, *SNCA*, which encodes the α-syn protein, stands out in research due to its association with forms of PD that follow an autosomal dominant inheritance pattern [38]. The presence of α-syn in Lewy bodies is a pathological hallmark of PD, prompting significant interest in understanding its pathogenic role [39].

The gut dysbiosis theory proposes that the accumulation and misfolding of α-syn in the gastrointestinal tract favor its aggregation and dissemination to the central nervous system (CNS) via the vagus nerve, starting in the brainstem, progressing to vulnerable areas such as the medulla oblongata, pons, and midbrain, and finally reaching the cerebral cortex. This process triggers a pathogenic cascade associated with neuroinflammation and the degeneration of various neuronal populations [44,45].

Indeed, analyses of gastrointestinal mucosal biopsies from PD patients corroborate the presence of an inflammatory process characterized by microglial cell activation and α-syn aggregates, which exacerbate neurodegeneration [46].

**Figure 1:**
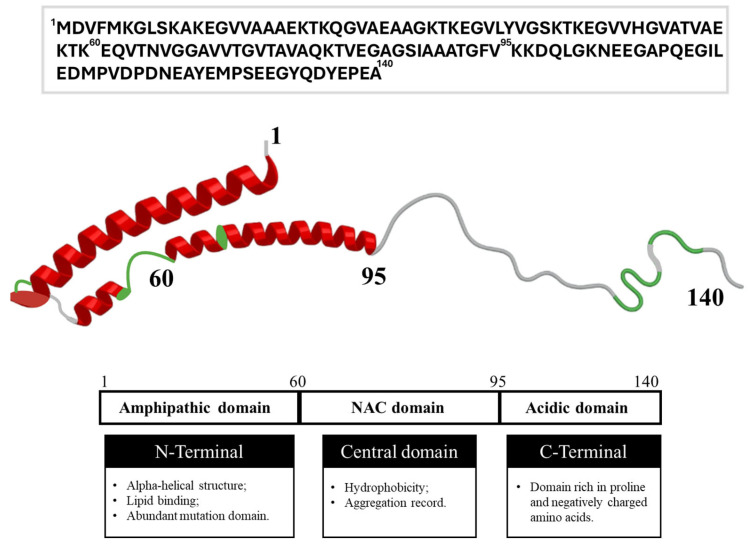
Structure of monomeric α-syn protein and its three main domains. The N-terminal region (1–60 aa) and the NAC region (61–95 aa) are predominantly represented as α-helical structures (red), with short loop segments (green) interspersed. The C-terminal region (96–140 aa) is depicted as an unstructured tail (grey) with flexible loops (green), consistent with high-resolution structural data from α-syn fibrils and ligand complexes (NCBI accession NP_001362214.1). The NAC region represents the principal aggregation-prone domain associated with oligomerization and fibril formation, whereas the N-terminal and C-terminal regions contain major epitopes explored in active and passive immunotherapeutic strategies. Structural domain organization is particularly relevant for rational epitope selection, influencing antibody specificity, aggregate selectivity, and therapeutic targeting of distinct α-syn conformations.

α-syn is recognized as a major contributor to the neuropathology of PD. This protein is highly expressed in the CNS, particularly at presynaptic terminals, where it participates in synaptic vesicle trafficking and neurotransmitter release [47,48]. α-syn belongs to the synuclein family, which also includes β- and γ-synucleins, and consists of a highly conserved 140-amino-acid sequence encoded by the *SNCA* gene [49]. Structurally, monomeric α-syn is commonly divided into three main domains (Figure 1) [47,48]. The N-terminal region (residues 1–60) is hydrophilic and positively charged, allowing membrane interaction through the formation of amphipathic α-helices enriched in imperfect KTKEGV repeats. Importantly, several familial PD-associated mutations, including A30P, E46K, and A53T, are located within this domain [48,50].

The central region (residues 61–95), known as the non-amyloid-β component (NAC) domain, is highly hydrophobic and plays a critical role in α-syn aggregation and fibril formation. Although originally identified in amyloid plaques from Alzheimer’s disease, the NAC region is now recognized as the principal aggregation-prone segment responsible for the amyloidogenic properties of α-syn [47,48]. The C-terminal domain (residues 96–140), often referred to as the acidic tail, is highly enriched in negatively charged residues and lacks a stable secondary structure. This region mediates interactions with proteins, metal ions, small molecules, and cellular membranes, including calcium-dependent interactions. In addition, the C-terminal domain is a major site of post-translational modifications, particularly phosphorylation at serine 129 (S129), which has been strongly associated with PD pathology [47,48].

Understanding the structural characteristics of α-syn domains is essential to elucidating their mechanisms of membrane interaction, pathological aggregation, and involvement in neurodegenerative diseases, especially PD. This knowledge contributes to the identification of therapeutic targets and to understanding the molecular processes underlying the formation of toxic aggregates [48,50].

At physiological pH, α-syn exhibits an uneven distribution of physicochemical properties along its amino acid sequence [51]. Specifically, the C-terminal domain contains a high proportion of acidic residues at physiological pH, suggesting a potential role in inhibiting α-syn aggregation. However, this inhibition of fibril formation can be altered when the pH shifts from neutral to acidic [52]. Additionally, this protein has attracted interest as a target for PD immunotherapies, making it a key therapeutic focus [53].

Although α-syn is one of the most extensively studied proteins today, particularly because of its association with PD, its physiological functions remain poorly understood. Some studies suggest that it may play an important role in stabilizing and modulating membrane structures, thereby regulating plasticity. It is also involved in membrane–membrane interactions at presynaptic nerve terminals [47,48].

**Figure 2:**
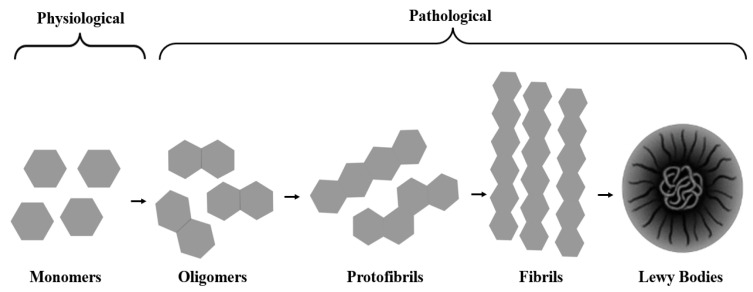
Multiple conformations of the α-syn protein. Illustrative representation of physiological (monomers) and aggregated α-syn conformations, including oligomers, protofibrils, fibrils, and Lewy body-associated deposits. Soluble oligomeric and protofibrillar species are widely considered the most neurotoxic conformations due to their involvement in synaptic dysfunction, membrane disruption, and neuronal injury, whereas mature fibrillar inclusions and Lewy bodies may additionally function as relative sinks for toxic intermediates, depending on the disease stage and cellular context. Several active and passive immunotherapeutic strategies preferentially target oligomeric or fibrillar α-syn species to reduce aggregation and limit the prion-like propagation of pathological α-syn between neuronal populations.

Under pathological conditions, α-syn aggregates form oligomers and fibrils that eventually assemble into Lewy bodies (Figure 2). Although Lewy bodies remain a pathological hallmark of PD, accumulating data suggest that small, soluble oligomeric intermediates are more directly linked to toxicity, whereas Lewy bodies may in part reflect a cellular attempt to sequester these species. This dual perspective needs to be considered when interpreting immunotherapeutic strategies that target aggregated α-syn forms [54,55,56,57,58,59].

## 3. Neuroinflammation in Parkinson’s Disease as an α-Syn Target: Proteomic Perspectives

Bidirectional communication between the brain and the immune system may facilitate the clearance of α-syn [60]. In the context of neurodegeneration-related neuroinflammation in PD, the innate immune system plays a crucial role, responding rapidly to attacks against misfolded α-syn, and constitutes a central molecular target for proteomic studies [61]. One of the main cells involved in this process is the microglia. These monocyte-lineage glial cells, when activated in the CNS, play a vital role in immunosurveillance. Their activation is protective and helps to remove α-syn aggregates, thereby promoting neuroprotection. However, chronic microgliosis can contribute to cytotoxicity and dopaminergic neuron death [62,63].

In addition to microglia, astrocytes and other glial cell types contribute to α-syn clearance and pathology propagation. Astrocytes internalize extracellular α-syn, modulate glutamate homeostasis, and release cytokines that can either support neuronal resilience or amplify neuroinflammation, depending on their activation state. Oligodendroglial engagement is particularly relevant in multiple system atrophy, but astroglial and microglial responses in PD share overlapping pathways, suggesting that α-syn-targeted immunotherapies may reshape a broader glial network rather than microglia alone [59,61,64].

Neuroinflammation and oxidative stress play distinct yet complementary roles in the pathophysiology of PD, with microglial activation as a central event [65]. In patients, activated microglia are observed in the substantia nigra, associated with the presence of pro-inflammatory factors in the brain and α-syn in the cerebrospinal fluid, while animal models induced by rotenone, MPTP, and 6-hydroxydopamine (6-OHDA) also highlight this inflammatory profile [66,67]. In this context, the recognition of α-syn by toll-like receptors (TLRs), such as toll-like receptor 2 (TLR-2) and toll-like receptor 4 (TLR-4), promotes the activation of the NF-κB pathway by phosphorylating and translocating nuclear factor kappa B, thereby amplifying the neuroinflammatory response in the disease [68].

Proteomic studies indicate that microglia activation is associated with a neurotoxic molecular profile, marked by the overexpression of Fyn kinase, heat shock proteins, and enzymes that produce reactive oxygen species. Fyn kinase mediates neuroinflammation in PD by activating the NOD-like receptor family pyrin domain containing 3 (NLRP3) inflammasome, a pattern recognition receptor (PRR). Resulting in the release of pro-inflammatory cytokines, such as interleukin-1 beta (IL-1β), involved in the inflammatory response and phagocytic clearance of cellular debris [69,70].

After activation by microglia, effector CD4^+^ T lymphocytes trigger an antigen-specific immune response and differentiate into pro-inflammatory subtypes, such as T helper 1 (Th1) cells, which secrete cytokines like interferon-gamma (IFNγ) and tumor necrosis factor alpha (TNF-α), and T helper 17 (Th17) cells, which secrete neurotoxic cytokines associated with neuronal death and the progression of PD. These cells release high levels of toxic cytokines, including interferon-gamma (IFNγ), interleukin-6 (IL-6), interleukin-12 (IL-12), and interleukin-17 (IL-17), highlighting their deleterious nature [71,72,73].

### 3.1. Vaccine Adjuvants with Neuroprotective Potential in Immunotherapy Against α-Syn

Adjuvants are substances added to vaccines to enhance and prolong the immune response, a concept introduced by Gaston Ramon in 1920 after observing that certain compounds, such as starch and inactivated diphtheria toxin derived from the bacterium *Corynebacterium diphtheriae*, induced local inflammation and increased antibody production. Subsequently, between 1925 and 1926, Alexander Thomas identified the adjuvant properties of the chemical compound alum (aluminum hydroxide). This discovery led to the development of an inactivated diphtheria toxin precipitated with alum [74,75].

Following these studies, aluminum-based adjuvants were widely incorporated into vaccines due to their ability to stimulate antibody production and reduce the need for additional doses [76,77]. In recent years, many studies have investigated new additives, especially natural compounds, to stimulate the innate and humoral immune system, exhibiting high immunomodulatory capacity, in addition to providing antigen-specific clonal expansion, promoting a potentiating adaptive immune response and increasing antigenic efficacy [78,79].

The species *Ananas comosus*, commonly known as pineapple, contains biomolecules of commercial interest, such as bromelain, a proteolytic enzyme found in plants of the *Bromeliaceae* family. This substance has demonstrated anti-inflammatory [80], antioxidant [81], anticancer [82], and cardioprotective properties [81,82]. In the context of complementary therapy, bromelain has been studied as an adjuvant in the treatment of inflammatory diseases with activity in modulating adhesion molecules on the surfaces of macrophages, CD4^+^ T cells, and natural killer cells [78,82].

Beyond the action of natural proteins, it is speculated that dendritic cell-based vaccines, which act as natural adjuvants, may positively influence the treatment of neurodegenerative disorders and be considered a promising strategy against PD [83,84]. These cells, when activated by antigens, can enhance the specific immune response of T helper 2 (Th2) cells, thereby stimulating antibody production. This process may contribute to the elimination of intracellular protein aggregates and favor an increase in regulatory T cell responses [85].

Potent adjuvants in vaccines, such as those used in Alzheimer’s studies, have been shown to induce Th2-type immune responses, favoring T lymphocyte differentiation and a more targeted response, regardless of the peptide used [85,86]. Furthermore, strategies such as dendritic cell vaccination can enhance the peripheral immune response in aging and represent a promising approach to modulate inflammation and protect dopaminergic neurons in PD [84,85].

Consequently, this approach may represent an effective therapeutic alternative, highlighting the need for further experimental studies on dendritic cell vaccination in PD [84,85]. Therefore, the addition of vaccine adjuvants is a promising strategy for developing new therapeutic approaches in the pharmaceutical industry to combat PD [86].

### 3.2. Structural Screening of α-Syn Ligand Receptors in the PDB for Parkinson’s Disease Therapies

The Protein Data Bank (PDB) (https://www.rcsb.org/) records provide a fundamental basis for the study of the molecular mechanisms of α-syn, allowing the integration of structural and proteomic data (Table 1). The available structures, obtained by X-ray crystallography, nuclear magnetic resonance (NMR), and cryo-electron microscopy, have broadened the understanding of α-syn toxicity and its relationship to PD, while emerging techniques, such as microelectron diffraction (MicroED), represent important advances in structural biology [21,22,87].

High-resolution structures deposited in the PDB (4RIK, 4RIL, and 4ZNN; up to 1.41 Å) allowed the identification of fundamental properties for understanding the behavior of full-length α-syn fibrils. A toxic segment of 11 residues (68–78), named NACore, whose atomic-resolution structure was determined by MicroED. The results revealed protofilaments formed by pairs of β-sheets with unusual characteristics, including the presence of water molecules at the interface. Comparative analyses demonstrated that NACore reproduces the structural and toxic properties of complete α-syn fibrils. In vitro assays indicated that NACore forms fibrils rapidly and exhibits high toxicity to PC12 cells, comparable to that of aggregated full-length α-syn, highlighting it as a promising target for the development of inhibitors of nucleation and growth of pathological fibrils [88].

The crystallographic structures of PDBs 8JJV and 8JLY, with resolutions of 1.23 Å and 1.29 Å, respectively, elucidate the interaction of the nanobody Nbα-syn01 with a 14-residue (43–56) peptide derived from α-syn. Structural analysis revealed a binding mode dominated by main-chain interactions, mediated by the CDR-H3 loop, whose binding site includes critical residues (48–52) associated with mutations in early-onset PD [89]. Deletion of the N-terminal region of the nanobody significantly increased the affinity for the peptide without altering its secondary structure. In vitro assays have demonstrated that both forms of the nanobody inhibit the formation of α-syn fibrils, with the truncated variant exhibiting greater efficacy, highlighting the potential of nanobody structural engineering as a therapeutic and diagnostic strategy to modulate pathological amyloid aggregation [89].

In line with these findings, recombinant antibody fragments, including nanobodies such as NbSyn2 and NbSyn87, bind to the highly exposed C-terminal region of α-syn and inhibit α-syn aggregation with high affinity, and are characterized as promising structural probes and potential immunotherapeutic tools in PD research [90].

Recent advances highlight the use of antibodies to selectively modulate α-syn. The antibody BIIB054 (cinpanemab), targeting N-terminal epitopes of α-syn (M1-K10; PDB 6CT7, 1.90 Å), has a higher affinity for N-terminally acetylated forms (~8-fold) and high selectivity for aggregates [91]. Characterization combined proteomic, biophysical, and structural approaches, including sequential fractionation of brain tissue, quantitative Western blot, enzyme-linked immunosorbent assay (ELISA), isothermal calorimetry, surface plasmon resonance, and crystallography. BIIB054 showed high affinity for fibrils (EC50~120 pM) and preferential detection of pathological species over monomers. In an in vivo model of PD, the treatment reduced the 6 kDa pathological fragment/total α-syn ratio by 30%, promoted functional improvement (50% in the wire-hang test), and preserved dopaminergic terminals, supporting its advancement to phase II clinical trials [91].

Concurrently, a structural and proteomic perspective, the selective recognition of α-syn by the monoclonal antibody MJFR14-6-4-2 (PDB 8OG0, 1.71 Å), identifies the C-terminal sequence EPEA (residues 137–140) as the minimal epitope, whose integrity is essential for binding. Analyses combining crystallography, NMR, and biochemical assays have shown that this epitope remains partially hidden in the monomeric form of the protein, becoming more exposed in oligomeric or fibrillar species. This greater structural exposure favors the antibody’s affinity for pathological aggregates. The insertion of an additional glycine at the C-terminal abolished recognition by MJFR14-6-4-2, allowing the generation of undetectable fibrils and improving the discrimination of endogenous aggregates in cellular models [92].

The interaction between cyclophilin A (CypA) and α-syn oligomers, as demonstrated by NMR and high-resolution X-ray crystallography (PDB 6I42, 1.38 Å), highlights the potential to modulate toxic aggregates. The study showed that CypA catalyzes the cis/trans isomerization of Pro128 in the acidic C-terminal domain of α-syn and additionally recognizes a high-affinity site in the hydrophobic PreNAC region (47–56). The pathogenic A53E mutation reduced PreNAC affinity, demonstrating that structural alterations in α-syn modulate its interaction with PPIases and impact amyloid aggregation, highlighting CypA as a promising therapeutic target in synucleinopathies [93].

In quantitative proteomics, sample preparation is crucial for the quality of results, especially for detecting low-abundance proteins, such as α-syn, whose analysis is limited by the wide dynamic range of proteins and ion quenching in mass spectrometry. Pre-analytical enrichment strategies are therefore essential to increase sensitivity in liquid chromatography-tandem mass spectrometry (LC-MS/MS), including protein precipitation, immunoprecipitation, direct digestion, and SDS gel fractionation, methods widely applied to the analysis of α-syn in biofluids [87,94,95].

These studies, which employ fibril and oligomer characterization through biological assays to obtain supramolecular structures at highly precise resolutions (<2.0 Å), provide insights into structural features and, when combined with large-scale proteomic analyses, allow mapping of molecular patterns associated with different α-syn conformations and therapeutic responses. This contributes to a better understanding of the mechanisms involved in PD. Thus, data obtained from the PDB are promising for the development of novel therapeutic approaches based on α-syn modulation, with potential to improve treatment strategies and clinical interventions [87,88,89,90,91,92,93,94].

### 3.3. Advances in the Development of α-Syn-Targeted Immunotherapies for Parkinson’s Disease

Since Edward Jenner developed the first vaccine in 1796, vaccination has played an essential role in preventing infectious diseases such as measles and rubella. Despite ongoing challenges, this strategy has been vital for controlling outbreaks, eliminating pathogens, and even eradicating diseases, as exemplified by smallpox [95]. Currently, evidence shows that vaccines combat more than 30 pathogens across roughly 70 regions worldwide, highlighting their broad efficacy and global impact on public health [96].

In recent years, the role of vaccines has expanded beyond prophylaxis, emerging as a therapeutic alternative for cancer and neurodegenerative diseases [97]. In particular, the search for rigorous and effective interventions for PD has intensified, aiming to preserve patients’ quality of life and slow disease progression [98]. In this context, immunotherapies, both passive and active, stand out as promising neuroprotective approaches.

α-syn has emerged as a central target in efforts to develop therapeutic vaccines for PD (Table 2). Evidence that T cell activation specific to this antigen is involved in disease pathogenesis, reinforcing its potential as a therapeutic target [99,100]. Therefore, targeting α-syn as a therapeutic immunization strategy may represent a significant advance in the treatment of this debilitating condition [52].

Active immunotherapies represent promising approaches for PD due to their safety profile and potential for early intervention. Phase I clinical trials with the PD01A, PD03A, and AFF 1 (AFFiRIS AG, Vienna, Austria) vaccines have demonstrated good tolerability and safety in patients [101,102,103].

These vaccines, called AFFITOPEs, use peptides from the C-terminal region of α-syn, conjugated to aluminum hydroxide, to induce a specific immune response and increase anti-α-syn antibodies. The PD01A vaccine, in doses of 15 µg and 75 µg, stands out for its selectivity for oligomers and significantly reduces α-syn levels in cerebrospinal fluid, demonstrating an impact on disease biomarkers. Despite limitations, such as the absence of a control group and the small number of participants, results with PD01A indicate a favorable safety and efficacy profile, with the vaccine showing potential to induce a sustained immune response [101].

The Swiss company AC Immune SA reformulated PD01A into ACI-7104, which uses a liposomal delivery platform to enhance immunogenicity. A phase II clinical trial, named VacSyn, is currently ongoing, recruiting participants with early-stage prodromal PD and documented α-syn pathology [104].

The PD03A vaccine candidate showed promising results, with functional improvements associated with reduced α-syn oligomers in key brain regions, including the basal ganglia (striatum and substantia nigra). In experimental models with Thy1*SNCA*/61 mice, immunization with PD03A improved functional performance, as measured by the wire-hang test. However, further studies are needed to confirm the influence of the Thy1 promoter on peripheral motor fibers [103].

The phase I study revealed that immunization with PD03A induced an immune response, with 88% of participants showing significant production of specific immunoglobulin G (IgG) antibodies, which were readily reactivated after booster doses. The safety profile was favorable, with mild, transient adverse events, such as erythema and swelling at the injection site. Although PD03A demonstrated good immunological potential, compared to PD01A, the immune response was more variable, with baseline antibody titers already elevated in some patients due to pre-existing antibodies in PD. Both candidates, PD01A and PD03A, were developed to mimic a critical epitope in the C-terminal region of α-syn, using amino acid substitutions to avoid autoimmune reactions, and both demonstrated safety and immunogenicity [101,103].

However, the study had limitations, such as a small sample size and a short duration. The results suggest benefits in patients with high levels of specific antibodies. Future phase II clinical trials, planned over 18 months, will be important for evaluating the clinical efficacy of PD03A, the persistence of the immune response, and the optimal intervals for booster doses. Preliminary data indicate that an annual dose may be sufficient to maintain therapeutic levels, consistent with results observed with PD01A [103].

The AFFITOPE AFF 1 vaccine has demonstrated efficacy in reducing α-syn oligomers and attenuating neurodegenerative deficits in experimental models of synucleinopathies, inducing a specific immune response through mimetic synthetic peptides, without cross-reactivity with other synucleins or induction of T-lymphocyte-mediated autoimmunity. The generated antibodies are directed to the C-terminal region of α-syn, favoring the recognition and clearance of toxic forms through mechanisms involving the internalization of antigen–antibody complexes and microglial activation, resulting in reduced pathology without adverse autoimmune effects. Additionally, immunization increased neuronal fractalkine levels, which were associated with decreased microgliosis and neuroinflammation, suggesting an anti-inflammatory effect mediated by the functional interaction between neurons and glial cells; however, the molecular mechanisms involved require further elucidation. However, the exact role of molecules such as fractalkine in this process remains to be investigated [102,103].

Preclinical datasets supported the advancement of the AFFITOPE platform into clinical development, marked by the initiation of phase I trials for the PD01A candidate. In α-syn-overexpressing models, AFF 1 administration induced significant antibody production clearable into the cerebrospinal fluid (CSF), plasma, and brain parenchyma. Notably, this was accompanied by a marked reduction in α-syn deposition and the preservation of striatal integrity, alongside the amelioration of cognitive and motor impairments, further reinforcing its therapeutic potential [101,102].

The human monoclonal antibody BIIB054 exhibits high selectivity for aggregated forms of α-syn, including recombinant fibrils. In initial clinical studies, doses up to 90 mg/kg were well tolerated, whereas 135 mg/kg was associated with adverse events, suggesting a safety limit at high doses. The 45 mg/kg dose demonstrated a safe profile and evidence of biological activity, with saturation of antibody binding to α-syn aggregates in cerebrospinal fluid, supporting the therapeutic potential of BIIB054; however, the evaluation of antibody-α-syn complexes still relies on ultrasensitive analytical methods [105,106].

Preliminary findings supported the clinical development of BIIB054 (cinpanemab) for synucleinopathies, although further refinement of sensitive pharmacodynamic biomarkers is still required to clarify its biological activity within the CNS [114]. In the phase II SPARK study involving patients with early PD (*n* = 357), cinpanemab administration for up to 112 weeks failed to produce significant improvements compared with placebo regarding clinical progression, neuroimaging parameters, or quality-of-life measures, ultimately resulting in study discontinuation following a planned interim analysis at week 72 [106]. The investigators emphasized a critical methodological limitation: the absence of validated approaches capable of reliably determining central target engagement and α-syn clearance during treatment. Accordingly, the clinical failure of these high-affinity anti-α-syn antibodies suggests that high binding affinity alone is insufficient to achieve disease modification. Several factors may account for this discrepancy, including the limited penetration of monoclonal antibodies across the BBB, the predominantly intracellular localization of pathogenic α-syn aggregates, and the substantial structural heterogeneity of α-syn species in human disease. Furthermore, many preclinical models rely on accelerated or artificial α-syn overexpression systems that incompletely recapitulate the progressive and multifactorial nature of PD. Clinical intervention may also occur too late in the neurodegenerative cascade, at a stage when synaptic dysfunction and neuronal loss are already well established and less biologically reversible. Collectively, these findings indicate that effective α-syn immunotherapy will likely require earlier intervention, optimized CNS target engagement, and therapeutic strategies capable of addressing the broader complexity of PD pathology.

In the Thy1*SNCA*/15 transgenic murine model, active immunotherapy with UB-312 demonstrated a good tolerability profile and robust immune response, without significant adverse effects. The treatment promoted selective reduction in α-syn oligomers without affecting monomeric forms, suggesting the potential to remove toxic species. A more pronounced effect was observed in the gastrointestinal tract than in the brain, suggesting therapeutic relevance for non-motor symptoms of PD [107].

Passive immunization with experimental antibodies directed against α-syn has been shown to inhibit the spread of this protein in animal models. Based on an in silico PK/PD model, the antibody MEDI1341 was designed to exhibit high affinity and efficacy at low concentrations, thereby reducing extracellular α-syn levels in the CNS. Despite limited penetration across the blood–brain barrier (BBB) (≈0.2–0.4%), MEDI1341 demonstrated the ability to reduce monomeric and fibrillar α-syn and to inhibit its spread in preclinical models, supporting its advancement to phase I clinical trials [108]. Concurrently, nanocarrier-based systems such as liposomes and polymeric nanoparticles are being investigated as complementary strategies to improve CNS delivery and target engagement. These platforms may enhance antibody stability, prolong systemic circulation, and facilitate BBB transit through surface functionalization and receptor-mediated transport mechanisms. Current evidence suggests that overcoming BBB-related pharmacokinetic limitations will likely require combined approaches integrating nanotechnology with molecular engineering and alternative brain-delivery strategies [98].

The phase I study with LU AF82422 evaluated the safety, tolerability, and pharmacokinetics of a single dose of the antibody in healthy individuals and Japanese PD patients. Results showed that LU AF82422 was well tolerated, with no serious adverse events or dose-limiting toxicity. The antibody displayed pharmacokinetic profiles consistent with expected IgG1 characteristics, including BBB crossing, with no CSF detection in all participants except one healthy individual. Exclusive administration of LU AF82422 resulted in a substantial reduction in free α-syn levels in PD patients, showing that α-syn reduction is well tolerated in individuals with synucleinopathies. Despite these positive results, short-term treatment did not induce a significant immune response. Additional studies with repeated doses are needed to explore long-term effects, with an ongoing study investigating the impact of repeated LU AF82422 doses in patients with multiple system atrophy. The antibody’s pharmacokinetics were linear and dose-proportional across doses ranging from 75 to 900 mg. However, some limitations were noted, such as the small number of participants and single-dose administration. Additionally, no adjustments were made for multiple comparisons in exploratory analyses. Overall, this single-dose study indicated that LU AF82422 is safe and well-tolerated, with a pharmacokinetic profile suitable for clinical development [109].

The authors demonstrated that early and prolonged administration of the mAb47 antibody, targeting α-syn oligomers and protofibrils in Thy1-h α-syn (A30P) transgenic mice, significantly reduced phosphorylated α-syn (pS129) deposits in the upper brainstem without altering total protein levels. The treatment showed positive, transient behavioral effects and did not induce neuroinflammation, demonstrating a favorable safety profile and potential for selective modulation of α-syn pathology in PD models [110].

The clinical study investigated the safety, tolerability, and pharmacokinetics of the experimental antibody Exidavnemab (BAN0805 or ABBV-0805) in healthy volunteers after a single administered dose. Results showed linear pharmacokinetics over the 100–6000 mg dose range, with consistent exposure profiles across Western, Caucasian, Japanese, and Chinese participants. Immunogenicity was low, with minimal incidence of anti-drug antibodies, and there was no significant impact on the antibody’s pharmacokinetics or safety. Exidavnemab stood out for its high affinity (KD = 18 pM) and 100,000-fold selectivity for aggregated forms of α-syn over monomers. Due to this high selectivity, the antibody showed potential to efficiently bind pathological α-syn aggregates in the CNS while preserving physiological monomeric forms.

A dose-dependent reduction in free plasma α-syn, predominantly consisting of monomeric forms, was observed, with maximal reduction achieved at doses of 1000 mg. The antibody elimination half-life was approximately 30 days, providing prolonged efficacy and continuous engagement with pathological α-syn aggregates. Regarding safety, Exidavnemab was well tolerated across all groups, with no signs of adverse effects related to administration. The excellent pharmacokinetic profile, combined with high selectivity and low immunogenicity, supports its therapeutic potential [111].

The phase II clinical trial evaluated the efficacy of Prasinezumab, a humanized monoclonal antibody targeting aggregated α-syn, in the treatment of PD. The study found no significant difference between Prasinezumab and placebo in disease progression after one year, as measured by the sum of Movement Disorder Society-Unified Parkinson’s Disease Rating Scale (MDS-UPDRS) Parts I, II, and III. Additionally, treatment had no detectable effect on nigrostriatal terminal degeneration, as assessed by SPECT imaging with 123I-ioflupane.

Evaluation of the primary endpoint was confounded by methodological limitations, specifically the improper censoring of data from participants who initiated concomitant dopaminergic or motor symptom medications. At week 52, about 30% of participants had their data censored, increasing to approximately 70% by week 104. Nevertheless, sensitivity analyses that included all participants confirmed the null results, reinforcing the robustness of the findings [112].

Other limitations of the study included the underrepresentation of non-white, non-American, or non-European populations, restricting the generalizability of the results to the global population with PD, in addition to the absence of specific tests to confirm the engagement of the therapeutic target, attributed to the lack of validated tools. In this context, prasinezumab, an antibody directed to the C-terminal region of monomeric and aggregated α-syn, demonstrated a favorable safety profile in patients with early-stage PD, although clinical and imaging outcomes did not show disease progression-modifying efficacy, reinforcing the need for future studies with improved methodologies and greater population diversity, as well as the inclusion of patients with confirmed α-syn pathology [112].

Accordingly, the use of daily active motor tests monitored by smartphones and smartwatches enabled continuous evaluation of motor parameters over two years, demonstrating significant potential for quantifying PD progression in early stages in individuals not yet treated with dopaminergic therapy [113].

Thus, although the results obtained so far are promising, the field remains in a consolidation phase, requiring synergy among basic research, clinical trials, and technological innovation to transform anti-α-syn immunotherapy from an experimental concept into an effective therapeutic reality in the fight against PD.

### 3.4. Patented Treatments Targeting α-Syn

Recent advances in proteomics and high-resolution structural data from the PDB have significantly deepened our understanding of α-syn pathology in PD, enabling the identification of immunogenic epitopes and specific therapeutic targets. In parallel, the number of patented strategies aimed at modulating α-syn has grown, including liposomal vaccines, dendritic cell-based immunization, and passive immunotherapy. However, there are still no approved vaccines for the treatment or prevention of the disease. Accordingly, this review examines four therapeutic approaches described in patents and evaluated in vivo for their effectiveness in treating PD (Table 3).

In 2023, AC Immune SA filed a patent application that evaluates a liposomal vaccine composition targeting the C-terminal region of α-syn. These liposome vesicles, composed of lipids, contain three key components: a peptide antigen derived from α-syn, a T-cell epitope, and an adjuvant [114]. The peptide sequence is specifically designed to include structural variations that enhance the immune response, without modifications that could compromise its efficacy. The T-cell epitope, crucial for inducing an adaptive immune response, and the adjuvant, which amplifies this response, are fundamental elements of the vaccine. The peptide sequence was carefully engineered to avoid inclusion of elements that could interfere with the immune response, such as the dipeptide YE at position X6. In preclinical tests, the liposomal vaccine sequence identification number (SEQ ID) NO:2 induced a more robust immune response in mice, with higher titers of specific antibodies against the peptide and α-syn, compared to the conjugate formulation. The liposomal formulation showed greater immunogenicity and efficacy, achieving a high response rate with a single dose, whereas the conjugate vaccine required two administrations, demonstrating the advantage of the liposomal strategy for immune induction. The results also indicate that the liposomal vaccine SEQ ID NO:2 may potentially play an important role in modulating the immune response against α-syn, a protein associated with neurodegenerative diseases such as PD. The vaccine aims to reduce the toxicity and aggregation of this protein, which is a key factor in the development of these conditions. The use of liposomes as a delivery vehicle for the peptide antigen enhances vaccine stability and bioavailability and potentiates T-cell activation, which is essential for an effective immune response. Furthermore, research suggests that co-delivery of the vaccine components, antigen and adjuvant, can improve immunogenicity [52,115]. In studies conducted in mice, the liposomal vaccine SEQ ID NO:2 induced higher antibody titers at all tested doses than the conjugated vaccine. Administration of the liposomal vaccine resulted in a robust immune response and superior control of α-syn aggregation, representing a significant advance in the research and development of therapeutic vaccines for neurodegenerative diseases. In this regard, these findings confirm the considerable potential of the liposomal vaccine SEQ ID NO:2 to induce an effective immune response against α-syn and its aggregated forms, suggesting it may be a promising candidate for the development of immunotherapies for diseases such as PD.

Another patent application, WO2022060487A1 [116], published in 2022 by Prothena Biosciences Limited, describes innovative peptide-based approaches and immunotherapies targeting α-syn, with potential application in synucleinopathies such as PD and dementia with Lewy bodies. The proposed strategies aim to reduce aggregation, remove deposits, and interrupt the propagation of α-syn, including the administration of peptide-encoding nucleic acids to allow controlled in vivo expression and a modifiable immune response as the disease progresses [116].

Complementarily, patent US20170196948A1 [117] (University of South Florida, 2017) described a vaccine based on dendritic cells sensitized with α-syn epitopes in a transgenic PD model (A53T), which induced a specific humoral response and improved motor deficits, without adverse inflammatory effects. Epitope analysis identified the C-terminal region as highly immunogenic, although no significant reduction in total brain α-syn levels was observed. Furthermore, the same invention explored passive immunotherapy with antibodies targeting distinct regions of α-syn, demonstrating that antibodies against the N-terminal were more effective in preventing neurodegeneration, improving behavior, and reducing microglial activation. Taken together, these findings reinforce the potential of active and passive immunotherapeutic strategies targeting specific α-syn epitopes as promising approaches for the treatment of PD [117].

Patent WO2009133521A2 [118], filed in 2009 by BioArctic Neuroscience Ab, describes an innovative therapeutic approach for disorders associated with α-syn, focusing on the modulation of its soluble oligomers. The aim is to delay the onset or treat α-syn-related diseases through a vaccine or specific antibodies that bind this protein in its soluble form. The key to this strategy lies in stabilizing α-syn oligomers, which exhibit a lower aggregation rate compared to unstabilized oligomers, minimizing the formation of insoluble species. The methodology involves immunizing Balb/C mice with α-syn protofibrillar or oligomeric specifications, modified with HNE (4-hydroxy-2-nonenal) or ONE (α,β-unsaturated 4-oxo-2-nonenal), using complete and incomplete Freund’s adjuvant. This immunization was repeated in multiple stages, with subsequent injections containing the planned modifications at final concentrations of 35 μM and 70 μM.

The immunization protocol was designed to induce a robust antibody response, which was tested using techniques such as ELISA to verify specific reactivity against modified α-syn oligomers. The polyclonal antibodies generated were tested for specificity and reactivity toward specific α-syn modifications. An ELISA analysis using microtiter plates coated with α-syn in different forms (monomeric, protofibrillar, or fibrillar) demonstrated that antibodies produced by immunized mice bound strongly to the modified forms of α-syn, particularly those containing derivatives HNE and ONE. In addition, one generated hybridoma (40:2) showed strong binding to ONE-modified α-syn, as evidenced by the analysis of its supernatant at various dilutions. These hybridomas represent a powerful tool for the development of antibody-based therapies, with potential applications in the treatment of α-syn-related diseases, such as PD [118].

The study described in the patent presents a therapeutic strategy based on vaccines and antibodies to modify the trajectory of α-syn-associated diseases, while also providing methods for detecting pathological forms of this protein. Thus, the impact and advantages of these approaches are further established as additional clinical trials targeting α-syn advance, aiming to mitigate the challenges posed by neurodegenerative diseases such as PD, which continue to represent a growing burden on public health.

**Table 3:** Inventions on immunizations against Parkinson’s disease targeting α-syn.

Code	Year of Filing	Patent Registration	Company	Inventor	Type of Immunization
WO2023152260	2023	Patent Cooperation Treaty (PCT)	Ac Immune SA, Lausanne, Switzerland [114]	Andréa Pfeifer (French)	Oligomers/Mice/Liposomal vaccine/Active immunization
WO2022060487A1	2022	Patent Cooperation Treaty (PCT)	Prothena Biosciences Limited, Dublin, Ireland [116]	Robin Barbour; Gene Kinney; Wagner Zago (French)	Literature review/Oligomers/Active immunization
US20170196948A1	2017	United States (USA)	Meganano Diagnostics Inc/University of Tampa, FL, USA [117]	Chuanhai Cao; Xiaoyang Lin (French)	Oligomers/Mice/Active immunization
WO2009133521A2	2009	Patent Cooperation Treaty (PCT)	Bioartic Neuroscience AB, Stockholm, Sweden [118]	Lars Lannfelt; Joakim Bergström; Martin Ingelsson (French)	Oligomers/Active immunization

## 4. Conclusions

Despite numerous advances in understanding the role of α-syn in PD, the development of effective therapies remains a significant challenge, particularly due to its polymorphic nature, the protein’s structural and functional complexity, its ability to propagate between dopaminergic neurons, and the heterogeneity of the aggregates formed. Moreover, the difficulty of drugs crossing the BBB compromises therapeutic efficacy in the CNS.

Recent evidence indicates that α-syn is not restricted to the CNS, being widely distributed in neural tissues and can propagate intercellularly among dopaminergic neurons, reinforcing its central role in the pathogenesis of synucleinopathies. In this context, immunotherapies targeting α-syn, including vaccines and high-affinity monoclonal antibodies, have emerged as promising strategies, with candidates such as PD01A, PD03A, AFFITOPE^®^ PD01A, ACI-7104, BIIB054, MEDI1341, and UB-312 demonstrating safety, immunogenicity, and preliminary neuroprotective potential in preclinical and early clinical studies. Nevertheless, clinical translation remains limited by methodological constraints, including small cohorts, challenges in biomarker monitoring, and variability in immune responses, highlighting the need for long-term, randomized clinical trials to establish sustained efficacy and safety.

The integration of proteomic, immunological, and in silico approaches represents a promising strategy for understanding the progression of PD, enabling the identification of molecular patterns associated with disease progression, predicting therapeutic responses, and optimizing vaccine protocols. Computational studies can accelerate epitope selection, improve strategies for removing toxic α-syn species, and contribute to the validation of early biomarkers with potential clinical applications.

The marked individual variability in therapeutic responses, particularly in advanced stages of the disease, underscores the need for more robust biomarkers that can be validated by proteomic analyses. In this context, integrated strategies based on genetic and molecular profiles, combined with bioinformatics tools, have become important approaches for developing more effective and personalized therapies for PD.

Clinical trials with monoclonal antibodies, such as prasinezumab, have demonstrated safety and encouraging signals in slowing disease progression. Additional interventions include aggregation-inhibiting peptides, which interfere with the formation of toxic oligomers, as well as approaches based on RNA interference and CRISPR-Cas9 aimed at modulating *SNCA* expression. Stimulation of autophagy and proteostasis mechanisms has also gained relevance, as it favors the degradation of protein aggregates. Furthermore, the use of nanocarriers such as liposomes, polymeric nanoparticles, and dendrimers has emerged as an innovative approach to overcome the BBB and promote controlled drug release in the CNS, thus increasing bioavailability and therapeutic specificity.

In summary, based on currently available evidence, α-syn-targeted immunotherapies highlight the urgent need for long-term, randomized preclinical and clinical studies that incorporate endpoints closely linked to mechanisms of action and α-syn-specific biomarkers, as well as careful patient stratification based on confirmed α-syn pathology and disease stage. Within this framework, the development of vaccines, antibodies, and nanobodies guided by structural and proteomic approaches, integrated with advanced in silico models and precision medicine strategies, appears to be the most promising path toward achieving truly disease-modifying immunotherapies in PD.

## Figures and Tables

**Table 1:** High-resolution structures (<2.0 Å) of α-syn bound to Homo sapiens molecules deposited in the Protein Data Bank (PDB).

PDB Code	Molecule (Receptor)	Ligand	Experimental Method	Resolution (Å)	Reference
4RIK	NACore	Fibril	X-ray diffraction	1.85 Å	[88]
4RIL	NACore	Fibril	Electron crystallography	1.43 Å	[88]
4ZNN	NACore	Fibril	Electron crystallography	1.41 Å	[88]
8JJV	Nbα-syn01	Fibril	X-ray diffraction	1.23 Å	[89]
8JLY	Nbα-syn01	Fibril	X-ray diffraction	1.29 Å	[89]
2X6M	Nanobody NbSyn2	Fibril	X-ray diffraction	1.62 Å	[90]
6CT7	Antibody BIIB054	Fibril	X-ray diffraction	1.90 Å	[91]
8OG0	Antibody MJFR14-6-4-2	Fibril	X-ray diffraction	1.71 Å	[92]
6I42	Cyclophilin A	Oligomers	X-ray diffraction	1.38 Å	[93]
9EUU	-	Fibril	Electron microscopy	1.93 Å	To be published

**Table 2:** Therapeutic immunization strategies under investigation for Parkinson’s disease targeting α-syn.

Immunotherapeutic	Type of Immunization	Conformation	Development Phase	Industry	Reference Number
AFFITOPE-PD01A	Active	Oligomeric	Phase I	AFFiRIS AG, Vienna, Austria	[101]
AFFITOPE-AFF 1	Active	Oligomeric	Phase I	AFFiRIS AG, Vienna, Austria	[102]
AFFITOPE-PD03A	Active	Oligomeric	Phase I	AFFiRIS AG, Vienna, Austria	[103]
ACI-7104/VacSYn	Active	Oligomeric	Phase II	AC Immune SA, Lausanne, Switzerland	[102,104]
BIIB054	Active	Fibrillar	Phase II	Biogen, Cambridge, MA, USA	[105]
Cinpanemab	Active	Fibrillar	Phase II	Biogen, Cambridge, MA, USA	[106]
UB-312	Active	Oligomeric/Fibrillar	Phase I	Vaxxinity/UBITh, Dallas, TX, USA	[107]
MEDI1341/Terepercimab	Passive	Monomeric	Phase I	AstraZeneca, Cambrige, UK	[108]
LU AF82422	Passive	Monomeric	Phase I	H. Lundbeck A/S, Copenhagen, Denmark	[109]
mAb47	Passive	Oligomeric/Protofibrillar	Phase I	BioArctic AB, Stockoholm, Sweden	[110]
ABBV-0805 (BAN0805)/Exidavnemab	Passive	Oligomeric/Protofibrillar	Phase I	BioArctic/AbbVie, North Chicago, IL, USA	[111]
PRX002/RG7935–Prasinezumab	Passive	Monomeric	Phase II	Prothena Bioscience, South San Francisco, CA, USA	[112,113]

## Data Availability

No new data were created or analyzed in this study. Data sharing is not applicable.

## Abbreviations

The following abbreviations are used in this manuscript:

HNE	4-hydroxy-2-nonenal
α-syn	α-synuclein
BBB	Blood–brain barrier
CSF	Cerebrospinal fluid
CNS	Central nervous system
CypA	Cyclophilin A
ELISA	Enzyme-linked immunosorbent assay
IFNγ	Interferon-gamma
IgG	Immunoglobulin G
IgG1	Immunoglobulin G1
IL-1β	Interleukin-1 beta
IL-6	Interleukin-6
IL-12	Interleukin-12
IL-17	Interleukin-17
LC-MS/MS	Liquid chromatography–tandem mass spectrometry
MDS-UPDRS	Movement Disorder Society-Unified Parkinson’s Disease Rating Scale
MicroED	Microelectron diffraction
MPTP	1-methyl-4-phenyl-1,2,3,6-tetrahydropyridine
NAC	Non-Amyloid Component
NF-κB	Nuclear factor kappa B
NLRP3	NOD-like receptor family pyrin domain containing 3
NMR	Nuclear magnetic resonance
PDB	Protein Data Bank
PD	Parkinson’s disease
PRR	Pattern recognition receptor
SEQ ID NO	Sequence identification number
SNpc	Substantia nigra pars compacta
Th1	T helper 1 cells
Th17	T helper 17 cells
Th2	T helper 2 cells
TLR-2	Toll-like receptor 2
TLR-4	Toll-like receptor 4
TLRs	Toll-like receptors
TNF-α	Tumor necrosis factor alpha
